# Machine learning-driven cancer diagnostics with improved robustness and interpretability

**DOI:** 10.1039/d6sc02368a

**Published:** 2026-06-02

**Authors:** Pengfei Li, Zhen Liu

**Affiliations:** a State Key Laboratory of Analytical Chemistry for Life Science, School of Chemistry, Nanjing University Nanjing 210023 Jiangsu China zhenliu@nju.edu.cn +86-25-8968-5639

## Abstract

Cancer remains one of the leading causes of death worldwide, underscoring the critical need for early diagnosis to improve long-term survival outcomes and reduce mortality rates. Despite significant progress, the development of effective cancer diagnostics continues to face two major challenges. First, the design and optimization of diagnostic assays and analytical workflows largely rely on empirical, trial-and-error approaches, which are inefficient and often yield limited robustness and generalizability. Second, the interpretation of high-dimensional and heterogeneous clinical imaging and molecular profiling data remains complicated, hindering interpretability and the translation of results into clinically actionable insights. Machine learning (ML), with its advanced capabilities in pattern recognition, optimization, and prediction, offers a promising approach to address both challenges for accelerating the development of next-generation cancer diagnostics. In this perspective, we briefly outline the widely used ML algorithms in cancer diagnostics and critically compare their strengths and limitations in real-world applications, considering factors such as data scale, class imbalance, feature structure, generalization performance, and model interpretability. We then summarize recent advances enabled by ML, ranging from analytical platform optimization to multiscale data interpretation. Finally, we discuss remaining challenges and propose a roadmap for future research in ML-driven cancer diagnostics.

## Abstract 2

This perspective introduces machine learning-driven approaches for cancer diagnostics and discusses how they enhance robustness and interpretability in assay optimization and multiscale data analysis.

## Graphical abstract

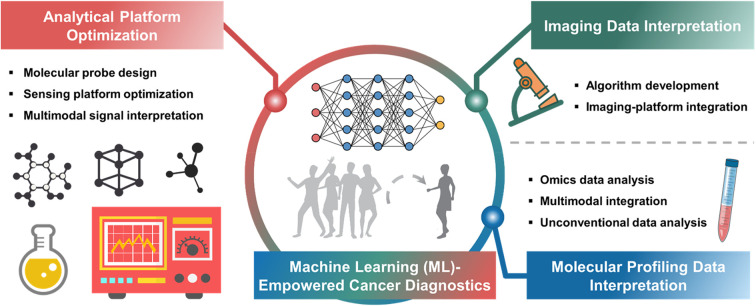

## 1 Introduction

Cancer is characterized by dysregulated cell growth, genomic instability, and the progressive acquisition of traits that support tissue invasion, immune evasion, and metastatic dissemination.^1,2^ It accounts for roughly 15% of all-cause mortality worldwide and remains a substantial global health burden.^3^ Early detection is essential because cancers identified at an initial stage are more responsive to curative treatment, leading to markedly improved survival outcomes and reduced therapeutic complexity. However, achieving effective early detection remains challenging. Many cancers are asymptomatic in their early phases, and validated biomarkers for early detection are still lacking for certain cancer types, such as triple-negative breast cancer.^4^ Furthermore, current diagnostic frameworks often rely on single biomarkers or narrowly defined molecular features, which are insufficient to capture the complexity, heterogeneity, and dynamic evolution of cancer biology and enable precise cancer diagnosis.^5^ For example, the widely used prostate cancer biomarker prostate-specific antigen (PSA) exhibits ethnicity-dependent baseline levels,^6^ indicating that a single fixed threshold applied across populations may contribute to overdiagnosis in groups with naturally higher PSA values. In addition, practical limitations such as the suboptimal sensitivity and specificity of many existing analytical assays further constrain the accuracy and reproducibility of current diagnostic tests.^7,8^ Consequently, there remains a persistent and unmet need for early cancer detection strategies with improved clinical utility.

To address these challenges, extensive efforts have been devoted to advancing cancer diagnostic technologies. These include the discovery of clinically relevant biomarkers through population-based multi-omics profiling,^9,10^ as well as the development of advanced imaging techniques and *in vitro* diagnostic assays.^11,12^ Collectively, these advances have substantially moved the field forward and helped narrow the gap between analytical validity, which evaluates how accurately and reliably a test measures biomarkers, and clinical validity, which assesses how well a test detects or predicts clinical diagnoses or outcomes.^13^ Nevertheless, the effective translation of these innovations into routine clinical diagnostics remains limited. A major challenge lies in the development and validation of diagnostic methods, which still rely heavily on empirical, trial-and-error optimization. For example, the performance of biosensor arrays, which are crucial for cancer diagnosis, depends critically on both the number and the quality of sensing elements.^14^ Combinatorial strategies can generate large libraries of candidate elements, but screening for optimal combinations with desirable cross-responsiveness often requires laborious manual experimentation that is time-consuming, resource-intensive, and poorly suited to capturing the biological complexity of cancer. Another major challenge arises from the interpretation of high-dimensional and heterogeneous clinical data. Molecular profiles derived from genomics, transcriptomics and proteomics contain rich but often subtle diagnostic signals that conventional statistical approaches struggle to extract or integrate across multiple modalities. Similarly, advanced imaging technologies generate vast quantities of data in which fine textural and structural features that are difficult to interpret using human assessment alone. As a result, much of the potentially valuable diagnostic information embedded in these datasets remains underutilized, creating a critical need for analytical approaches capable of efficiently extracting and analysing complex diagnostic signals.

Machine learning (ML) represents a rapidly evolving field that enables computational systems to learn from data and improve performance without explicit, hand-crafted programming.^15^ It has become integral to diverse sectors such as healthcare,^16^ finance,^17^ and material science.^18^ In the context of cancer diagnostics, numerous studies have demonstrated that ML can substantially enhance diagnostic accuracy, reproducibility, and scalability by optimizing assay parameters,^19^ integrating multi-omics datasets,^20^ and improving image interpretation.^21^ These capabilities position ML as a powerful tool for addressing long-standing gaps in early cancer detection and supporting more precise, personalized disease management. Despite this progress, translating ML-based approaches into reliable clinical diagnostics remains an open problem. In particular, during analytical method development and assay optimization, dataset bias often arises from systematic differences introduced by platform-to-platform variation, reagent batch effects, and sample preparation or acquisition workflows, which can cause models to learn technical signatures rather than true disease-related patterns. Some reported models are developed or evaluated using datasets that are limited in size, diversity, or clinical representativeness,^22–25^ which may further exacerbate this problem by making such bias harder to detect and correct, ultimately leading to biased performance and restricted generalizability. In addition, insufficient transparency in model design and validation often hinders reproducibility and clinical interpretability, especially when the measured signal is already influenced by matrix effects and signal noise, making it difficult to distinguish biologically meaningful features from experimental artifacts. Therefore, further efforts are still required to effectively integrate ML into cancer diagnostics.

**Fig. 1:**
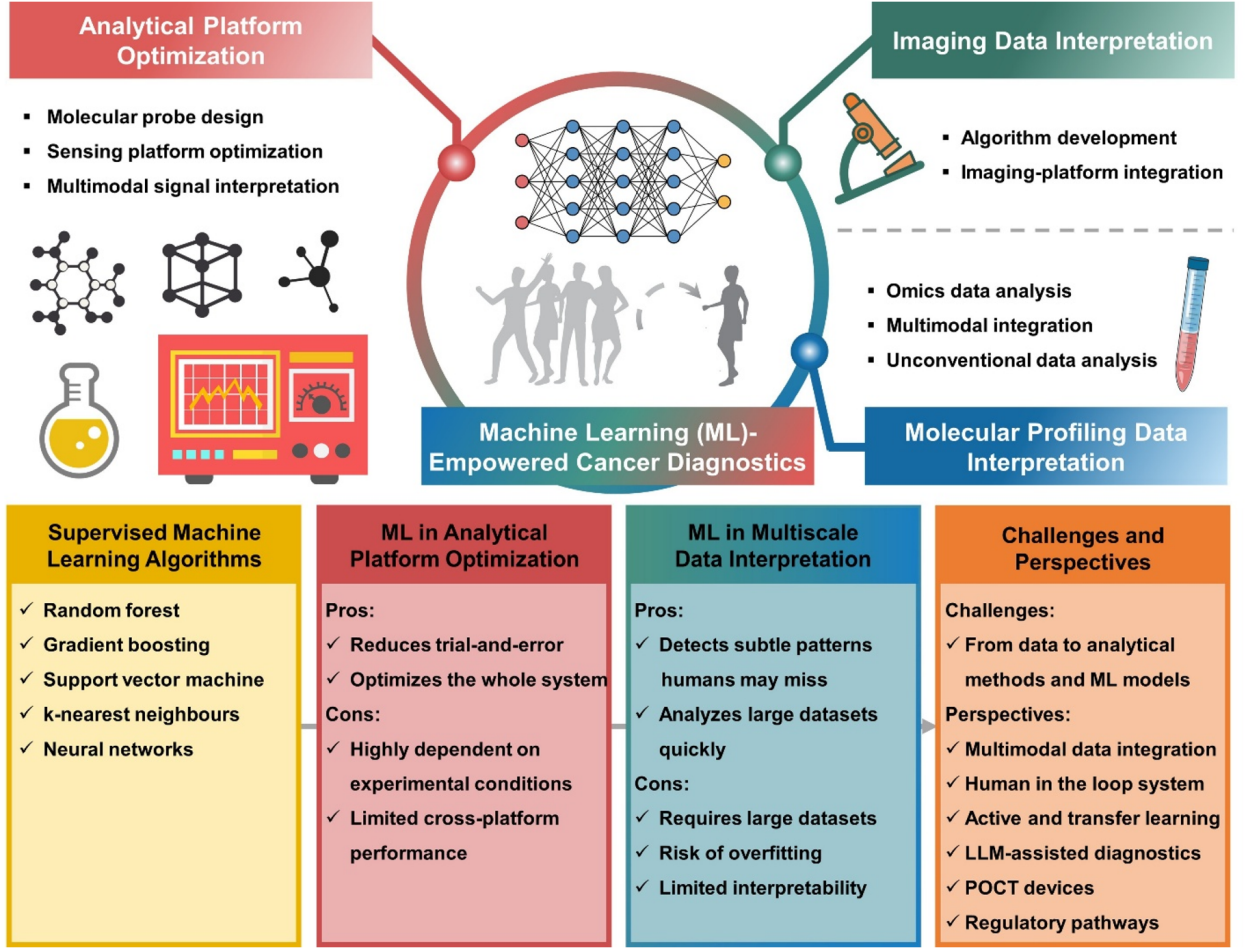
Overview of ML-based cancer diagnostics. Supervised ML algorithms are widely used due to their direct relevance to clinically interpretable prediction tasks and their suitability for both methodological optimization and clinical data analysis. Real-world applications of ML in cancer diagnostics are summarized and discussed with respect to their respective advantages and limitations. Major challenges and future perspectives are also highlighted to promote more reliable, robust, and clinically actionable performance in the integration of ML into cancer diagnostics.

Although several recent reviews have discussed ML in cancer research and diagnostics,^26–30^ they largely emphasize broad clinical applications, algorithmic comparisons, or imaging-based diagnostic pipelines. By contrast, the role of ML from an analytical chemistry perspective, particularly in analytical method development, assay optimization, and the interpretation of molecular profiling data, has received comparatively limited attention. Addressing this gap is essential, as many diagnostic bottlenecks arise at the level of analytical methods design and data interpretation rather than in downstream clinical application. In this perspective (Fig. 1), we aim to provide foundational knowledge on ML, with an emphasis on supervised ML algorithms due to their widespread use and strong capabilities in addressing cancer diagnostic challenges. We then highlight emerging applications of ML across the cancer diagnostics pipeline, including the design and optimization of diagnostic materials, detection platforms, and analytical workflows, as well as the processing and interpretation of imaging and molecular data. Rather than offering a comprehensive review of the literature, we selectively discuss representative studies to illustrate key concepts and to emphasize the potential opportunities for integrating ML into analytical and diagnostic development. Finally, we outline key challenges and future directions to guide the development of more robust, interpretable, and clinically actionable ML-enabled diagnostic systems.

## 2 Machine learning algorithms

ML is a data-driven subset of artificial intelligence that focuses on learning predictive patterns from data to support complex analytical tasks,^15^ and it has become the dominant analytical framework in cancer diagnosis. Within ML, deep learning (DL) has emerged as a prominent subfield and has advanced rapidly in recent years, particularly in medical imaging applications, such as lesion detection^31^ and segmentation.^32^ However, DL methods typically require large, well-annotated datasets, substantial computational resources, and extensive validation, which limits their practicality in some diagnostic scenarios, especially those involving data-limited cohorts or heterogeneous tabular features. For example, in small-sample omics studies and diagnostic classification tasks based on tabular features such as age, sex, and histopathological grade, traditional ML methods are often easier to regularize, less prone to overfitting, and better suited to high-dimensional but weakly structured data.^33^ Accordingly, this perspective is organized around the broader ML framework, with traditional ML approaches serving as the main focus. DL is discussed as an important component of ML rather than as the central scope of this perspective.

**Fig. 2:**
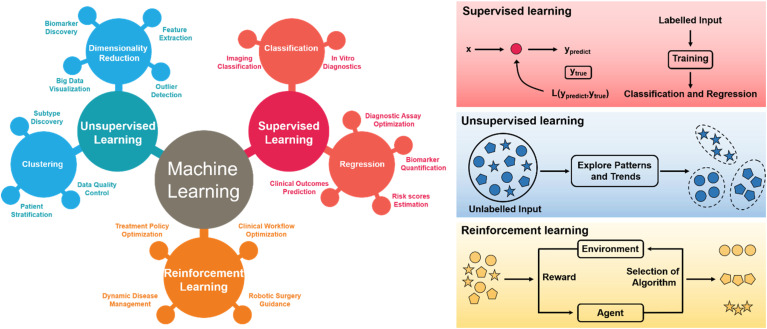
Classification of ML approaches (supervised, unsupervised, and reinforcement learning) according to the nature of supervision during training, and their representative clinical applications.

Based on the nature of the supervision available during training, that is, how and whether feedback is provided to the model, ML methods are commonly categorized into supervised learning, unsupervised learning, and reinforcement learning (Fig. 2). Supervised learning relies on explicitly labelled training data consisting of paired input–output examples, and models are trained by minimizing the discrepancy between predicted outputs and ground-truth labels through sample-level supervision.^34^ This paradigm is primarily applied to regression tasks, which aim to predict continuous-valued outcomes (*e.g.*, diagnostic assay optimization), and classification tasks, which assign samples to discrete categories (*e.g.*, benign *versus* malignant). In contrast, unsupervised learning operates on unlabelled data and does not assume the existence of predefined target variables. Instead, it seeks to uncover intrinsic structures, patterns, or representations by exploiting the statistical properties of the data.^35^ Common unsupervised learning tasks include clustering, which groups samples with similar characteristics, and dimensionality reduction, which maps high-dimensional data into lower-dimensional representations to improve interpretability and facilitate visualization. Common methods for these tasks include principal component analysis (PCA), hierarchical clustering, and *t*-distributed stochastic neighbour embedding (*t*-SNE).^36^ Among them, PCA is widely used to compress high-dimensional data while preserving the major variance structure, thereby supporting feature extraction and visualization, whereas clustering methods organize samples according to similarity and can help reveal latent group structure, such as potential biomarkers or previously unrecognized disease subtypes. *t*-SNE further facilitates the visualization of complex datasets by preserving local similarity relationships in low-dimensional space. As such, unsupervised learning is widely used for feature extraction, outlier detection, and data quality control in large-scale biomedical datasets. Reinforcement learning differs fundamentally from both supervised and unsupervised learning in that its learning signal is derived from delayed and often sparse reward feedback obtained through interaction with an environment, with the objective of maximizing long-term cumulative return in a sequential decision-making setting.^37^ Although reinforcement learning has shown promise in treatment policy planning and clinical workflow optimization, it remains less commonly applied in routine cancer diagnosis. Some literature additionally distinguishes semi-supervised learning, which leverages a small amount of labelled data together with a large pool of unlabelled data.^38^ However, as this paradigm does not introduce a fundamentally new form of supervision and is more appropriately viewed as an extension of supervised learning, it is not treated as a separate category in this perspective.

Given its direct relevance to clinically interpretable prediction tasks and its suitability for both methodological optimization and clinical data analysis, supervised ML has become the predominant paradigm in cancer diagnostic research. This prominence is largely attributable to its flexibility, robustness to heterogeneous biomedical data, and well-established performance across diverse cancer diagnostic applications, even under limited-data conditions. Importantly, the choice of supervised ML algorithms in cancer diagnostics is not determined solely by algorithmic sophistication, but is instead guided by the characteristics of the data and the clinical task. This section therefore focuses on representative supervised ML models that support both classification and regression tasks, including random forests (RF), gradient boosting trees (GBT), support vector machines (SVM), *k*-nearest neighbours (*k*NN), and neural networks (NN), and discusses their suitability using a set of practical factors that commonly guide model selection in cancer diagnostic studies.

Specifically, model selection in cancer diagnostics involves several key considerations: (1) Data scale: the balance between sample size (*n*) and feature dimensionality (*p*) is a critical factor. Cancer diagnostic studies often involve small patient cohorts but extract a large number of features, especially in omics-based analyses. This “small-*n*, large-*p*” problem increases the risk of overfitting, unstable parameter estimation, and limited generalization, and therefore directly influences whether highly flexible or more regularized models are appropriate; (2) Class distribution: in many diagnostic and screening scenarios, cancer cases represent a small minority relative to healthy controls. Such class imbalance determines whether standard training objectives are sufficient or whether algorithms that support class weighting or cost-sensitive learning are required; (3) Feature structure: the presence of redundant or highly correlated predictors, can reduce model stability and interpretability. Consequently, models that are robust to correlated features, which are common in omics datasets, are typically preferred; (4) Generalization performance: a clinically useful model must exhibit stability against data heterogeneity (*e.g.*, multi-centre designs or batch effects) and demonstrate consistent predictive performance across independent validation cohorts. This stability, evidenced by reproducible accuracy in diverse external datasets, is essential as it mitigates the risks of overfitting and systematic bias, thereby ensuring the model's reliability for real-world deployment; (5) Model interpretability: clinician acceptance is critical, as practitioners need to understand and trust a model's predictions. Therefore, the ability to explain individual predictions, identify key biomarkers, and align with clinical reasoning is essential for integrating models into diagnostic workflows and meeting regulatory expectations in healthcare. Different supervised ML algorithms exhibit distinct sensitivities to these criteria, and the relative importance of each consideration often determines the suitability of a given model for a specific cancer diagnostic task. These criteria therefore provide the conceptual basis for the comparative discussion of the five supervised ML methods reviewed in the following sections.

### 2.1 Random forest

**Fig. 3:**
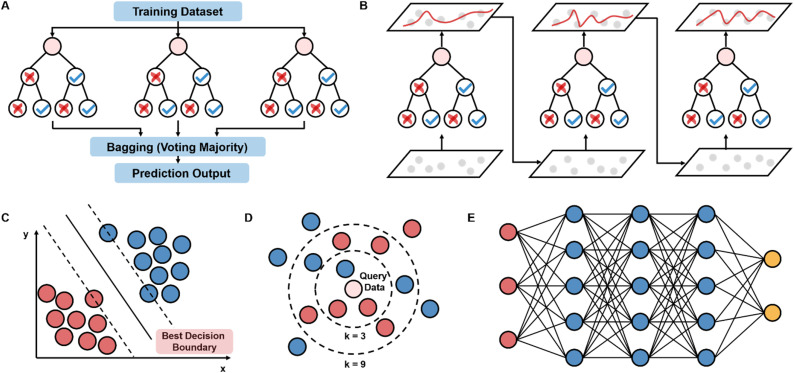
Representative supervised ML models that support both classification and regression tasks, including random forests (A), gradient boosting trees (B), support vector machines (C), *k*-nearest neighbours (D), and neural networks (E).

RF is an ensemble-based algorithm that constructs multiple decision trees during training (Fig. 3A). It is based on the principle of bootstrap aggregating, whereby each tree is trained on a bootstrap sample drawn with replacement from the original dataset, and at each split only a randomly selected subset of features is evaluated. The algorithm can be applied to both classification and regression tasks, with final predictions obtained by majority voting or by averaging, respectively. By combining the outputs of many decorrelated trees, RF effectively mitigates the high variance and overfitting that commonly affect individual decision trees, thereby transforming multiple weak learners into a single robust model with strong generalization capability.

Several properties of RF make it particularly well suited for cancer diagnostics. First, its ensemble structure leads to robust performance on unseen data by reducing prediction variance. This robustness can be assessed internally using the out of bag error, which is calculated from samples excluded during bootstrap sampling.^39,40^ In addition, RF performs well in the “small-*n*, large-*p*” setting, as randomized feature selection at each split acts as an implicit regularization mechanism that reduces dependence on any single predictor and limits overfitting.^41^ Owing to these characteristics, RF has been successfully applied to glioma grading using multiparametric magnetic resonance imaging radiomics.^42^ Furthermore, the algorithm is tolerant of correlated and redundant predictors, which reduces the need for extensive feature preprocessing while preserving potentially meaningful biological information.^41,43^

Despite these advantages, several limitations must be carefully considered in clinical applications. RF is sensitive to severe class imbalance, as the standard algorithm optimizes overall accuracy and may therefore favour the majority class, resulting in reduced sensitivity to minority class samples. To mitigate this limitation, balanced RF variants or the assignment of higher-class weights to the minority class during training are typically required.^44^ Another important challenge concerns interpretability. RF provides a degree of interpretability through feature importance measures, such as mean decrease in Gini impurity or accuracy, which can highlight potential biomarkers. For example, these measures can help identify discriminatory metabolites associated with prostate cancer, thereby guiding subsequent biological investigations.^45^ However, the ensemble model itself remains a black box and cannot generate a single, transparent decision rule to explain individual predictions in the way that a single decision tree can. Finally, although RF is relatively robust to random noise, its performance may deteriorate in the presence of systematic data heterogeneity, such as batch effects arising from differences in imaging protocols or sample processing across institutions. Consequently, careful data harmonization and rigorous external validation using independent multicentre cohorts are usually necessary before clinical translation.

### 2.2 Gradient boosting trees

GBT is a powerful ensemble-based algorithm that constructs predictive models in a sequential, stage-wise manner (Fig. 3B). Unlike the parallel learning strategy employed by RF, GBT builds a series of weak learners, typically shallow decision trees, in which each new tree is trained to correct the residual errors of the combined ensemble formed by all preceding trees.^46^ This learning process is formalized as the optimization of a differentiable loss function, such as log loss for classification or squared error for regression, using gradient descent. The final model generates predictions as a weighted sum of the outputs from all trees in the sequence.

Several highly influential software libraries, including XGBoost,^47^ LightGBM,^48^ and CatBoost,^49^ have been developed to implement and substantially enhance the core GBT framework. One of their most critical advancements is the explicit incorporation of regularization techniques. These techniques penalize excessive model complexity, thereby reducing overfitting and improving generalization to unseen data. This controlled learning framework enables modern GBT to capture complex, non-linear patterns and higher-order feature interactions with high accuracy, frequently outperforming alternative algorithms in both classification and regression tasks. In addition, GBT natively supports sophisticated handling of class imbalance through built-in sample weighting and cost-sensitive loss functions,^46,47^ making it particularly well suited for early detection studies in which cancer cases are rare.

Despite these strengths, the application of GBT in clinical research presents several notable challenges that require careful methodological oversight. A primary concern is its pronounced susceptibility to overfitting, particularly in small-sample settings.^50^ This risk is further exacerbated when features are highly correlated and regularization is insufficient. Consequently, disciplined training strategies are essential, including early stopping based on validation performance, low learning rates, and strict constraints on tree depth and node splitting.^47^ Moreover, GBT models are computationally intensive and highly sensitive to hyperparameter configuration.^51^ Achieving optimal performance depends on careful tuning of parameters such as the number of trees, learning rate, subsampling ratio, and regularization coefficients, often necessitating extensive cross-validation and substantial computational resources. Most critically, GBT models exhibit limited interpretability and are frequently considered even more opaque than RF. Although global feature importance measures, such as gain-based importance, are available,^47,52^ they provide limited insight into the decision logic underlying individual predictions. This lack of transparency poses a significant barrier to clinical adoption. Finally, similar to RF, while GBT is relatively robust to random noise within a stable data distribution, its performance can also deteriorate substantially in the presence of systematic data heterogeneity, such as multi-centre batch effects.

### 2.3 Support vector machine

SVM are supervised ML algorithms rooted in statistical learning theory and structural risk minimization (Fig. 3C). Their primary objective is to identify an optimal decision boundary, defined as a hyperplane, that separates data from different classes while maximizing the margin between them in the feature space.^53^ When the data are not linearly separable, SVM employ kernel functions to implicitly map the input data into a higher-dimensional space in which linear separation becomes feasible. Commonly used kernels include linear, polynomial, and radial basis function (RBF) kernels.

This margin-maximization framework allows SVM to model complex, nonlinear decision boundaries while maintaining strong generalization performance. As a result, SVM are particularly well suited to the “small-*n*, large-*p*” applications, especially in genomic biomarker-based cancer diagnosis.^54^

Despite their advantages, the practical deployment of SVM presents several important limitations that require careful consideration. One major challenge is sensitivity to class imbalance. The standard maximum-margin formulation assigns equal importance to all training samples, which can bias the decision boundary toward the majority class in screening or early detection contexts. Class-weighted SVM,^55^ which impose higher penalties for misclassifying minority class instances such as true cancer cases, are commonly used to address this issue. However, even with weighting, performance gains may be limited, particularly under conditions of severe imbalance. Additionally, SVM performance is highly dependent on appropriate feature scaling.^56^ Input variables generally require normalization, such as *Z*-score standardization, to prevent features with larger numeric ranges from disproportionately influencing the optimization process. SVM are also sensitive to outliers and noisy measurements, as these can significantly affect margin placement. This sensitivity necessitates rigorous data cleaning and preprocessing, especially when integrating heterogeneous, multi-centre clinical datasets. Finally, model interpretability remains a significant limitation of SVM, particularly when nonlinear kernels such as the RBF kernel are used. To mitigate this limitation, post hoc explanation techniques are often required to provide insight into model predictions.

### 2.4 *k*-Nearest neighbours

*k*NN algorithm is a simple, instance-based, non-parametric method used for both classification and regression tasks in cancer diagnostics (Fig. 3D). Its core principle is that samples with similar feature profiles tend to belong to the same class or exhibit similar output values.^57^ In classification tasks, the label of a new sample is determined by a majority vote among its *k* closest training samples in the feature space, as measured by a distance metric such as Euclidean distance. In regression tasks, predictions are typically obtained by averaging the target values of these *k* nearest neighbours.

This method possesses distinct advantages and disadvantages that determine its suitability for cancer diagnostics. Its primary strength is exceptional interpretability. The logic of “a patient is diagnosed in the same way as the *k* most phenotypically or molecularly similar patients in the cohort” is intuitively clear to clinicians, making model decisions easy to communicate. However, *k*NN suffers from poor computational efficiency at inference time.^58^ Because the algorithm requires computing distances between a query sample and all training samples, both prediction time and memory requirements scale linearly with dataset size, rendering *k*NN impractical for large-scale or real-time clinical applications. In addition, *k*NN is highly susceptible to the curse of dimensionality. In high-dimensional spaces, distance metrics become less meaningful, as all points tend to be equally far apart, leading to rapidly deteriorating performance. What's more, the algorithm is sensitive to noise and data structure. Its performance depends strongly on the choice of *k*, feature scaling, and the presence of irrelevant or correlated variables, such that even minor data heterogeneity can substantially alter nearest-neighbour relationships.^58^ Finally, *k*NN handles class imbalance poorly. In screening scenarios where healthy controls vastly outnumber cancer cases, the local neighbourhood of a rare positive sample is likely to be dominated by majority-class instances, biasing predictions toward the prevalent class.

### 2.5 Neural networks

NN are powerful computational models inspired by biological neural systems, consisting of interconnected artificial neurons arranged in layers (Fig. 3E). In this section, we focus on conventional feedforward NNs, which typically include an input layer, one or more hidden layers, and an output layer. Through iterative forward propagation and error backpropagation, NN adjust their internal weights to learn non-linear relationships directly from raw input data. This end-to-end learning capability reduces reliance on predefined feature structures, making NN particularly well suited for modelling complex associations in high-dimensional and structurally complex datasets.^59^ Furthermore, their architectures can be flexibly tailored to address specific clinical objectives. For example, specialized loss functions such as focal loss can be employed to impose higher penalties on misclassification of rare cancer subtypes,^60^ thereby improving predictive performance in class-imbalanced settings.

Despite these advantages, conventional NNs present substantial practical challenges. A major limitation is their strong dependence on large sample sizes.^28^ Robust training typically requires a number of samples that substantially exceeds the number of features; consequently, in omics studies where the number of features often far exceeds the number of patients, NN are highly susceptible to overfitting unless stringent regularization strategies or external datasets are incorporated. In addition, NNs are often regarded as “black-box” models,^61^ as their learned representations and decision mechanisms are not readily interpretable in clinically meaningful terms. This lack of transparency can undermine clinician trust and complicate model validation in high-stakes diagnostic contexts. Finally, NN training is sensitive to weight initialization, hyperparameter choices, and data distribution, which can lead to variability in performance across training runs and raise concerns regarding reproducibility and robustness, particularly in multi-centre studies.^62^

With the development of modern DL, more advanced architectures have emerged, including convolutional neural networks, recurrent neural networks, and transformer-based models.^63,64^ These architectures share some of the advantages and limitations of conventional NNs described above; however, compared with conventional NNs, they generally require larger datasets and greater computational resources while offering stronger feature-learning capacity in appropriate settings.

### 2.6 Comparison and applicable scope

As summarized in Table 1, there is no universally optimal algorithm for cancer diagnosis. RF achieve a favourable balance among robustness, ease of use, and a moderate degree of interpretability, and are therefore commonly employed as first-line exploratory approaches. SVM are particularly effective in settings with extremely small sample sizes, while GBT methods may be preferable when sufficient data are available and maximizing predictive performance is the primary objective. When model transparency and clinical persuasiveness are paramount, simpler and more interpretable approaches, such as *k*NN may offer distinct advantages. NN, by contrast, demonstrate irreplaceable value in specific domains such as medical imaging analysis; however, they also entail the highest computational, data, and implementation costs.

**Table 1:** Comparison and applicable scope of random forests (RF), gradient boosting trees (GBT), support vector machines (SVM), *k*-nearest neighbours (*k*NN), and neural networks (NN). Star ratings indicate relative strength, with ★★★★★ denoting the strongest and ★☆☆☆☆ denoting the weakest

	Data scale	Class distribution	Feature structure	Generalization performance	Model interpretability	Typical applications
RF	★★★★☆	★★★☆☆	★★★★☆	★★★★☆	★★★☆☆	Glioma grading using multiparametric magnetic resonance imaging radiomics^42^
	Performs well in the “small-*n*, large-*p*” setting, as randomized feature selection at each split reduces dependence on any single predictor and limits overfitting^41^	Supports balanced RF variants and class weights. But may favour the majority class by default^44^	Tolerates correlated and redundant predictors^41,43^	Provides a useful generalization estimate *via* out-of-bag error^39,40^	Offers feature importance for interpretability, but remains a black box for individual predictions	
GBT	★★★☆☆	★★★★☆	★★★☆☆	★★★☆☆	★★☆☆☆	Bladder cancer diagnosis by analysing atomic force microscopy images of cell surface structures^75^
	Performs well on medium to large datasets, but tend to overfit on small data; early stopping helps^47,50^	Handles imbalance well through sample weighting and cost-sensitive loss functions^46,47^	May overfit when features are highly correlated and regularization is weak^51^	May generalize poorly when hyper-parameters are not well tuned	Offer limited interpretability compared with RF; feature importance is available but limited^47,52^	
SVM	★★★★★	★★☆☆☆	★★☆☆☆	★★★★☆	★★☆☆☆	Pathological diagnosis across multiple cancer types using high contrast fluorescence imaging^76^
	Serve as a classic choice for “small-*n*, large-*p*” setting due to the margin-maximization framework^54^	Perform poorly with class imbalance and usually require class-weighted methods^55^	Sensitive to feature scaling, outliers, and noise^56^	Offer good theoretical generalization, but depend strongly on kernel and parameter choices	Hard to interpret, especially with kernel methods	
*k*NN	★★☆☆☆	★☆☆☆☆	★☆☆☆☆	★★☆☆☆	★★★★☆	Cancer biomarker p53 detection *via* voltametric features extraction^68^
	Impractical for larger-scale data^58^	Suffer from majority-class dominance in local neighbourhoods	Suffers from the curse of dimensionality	Sensitive to noise and local structure^58^	Very intuitive: “patients most similar to this one tend to suffer from the same disease”	
NN	★★☆☆☆	★★★★☆	★★★★★	★★☆☆☆	★☆☆☆☆	Accurate differentiation of tumour and adjacent normal tissues using mass spectrometry imaging^78^
	Require large datasets and easily overfit on small datasets^28^	Handle imbalance well with flexible losses such as focal loss^60^	Learn hierarchical feature representations automatically	Sensitive to weight initialization, hyperparameter choices, and data distribution^62^	Remain a black-box model^61^	

## 3 Application of machine learning in cancer diagnostics

Accurate cancer diagnosis is fundamental to clinical decision-making, therapeutic stratification, and outcome prediction; however, traditional diagnostic strategies that rely on single biomarkers or performance-limited measurements often fail to capture tumour heterogeneity and disease dynamics. In response, ML has emerged as a powerful enabler across the diagnostic workflow, reshaping both the development of diagnostic methodologies and the interpretation of increasingly complex diagnostic outputs. Accordingly, recent advances in ML-assisted cancer diagnostics can be broadly categorized into two interconnected domains: the optimization of diagnostic assays and analytical platforms, and the analysis of high-dimensional imaging and molecular profiling data. Collectively, these developments reflect a paradigm shift in cancer diagnostics from limited biomarker detection and performance-constrained designs toward data-driven frameworks that integrate diverse information layers to support accurate, scalable, and clinically actionable diagnosis.

### 3.1 Optimization of analytical procedures and detection platforms

The development and validation of diagnostic methodologies play a central role in cancer diagnostics, as they determine how diagnostic signals are acquired, quantified, and interpreted, ultimately shaping specificity, robustness, and clinical reliability. In recent years, ML has assumed an increasingly systematic role in molecular probe design and optimization, the construction of analytical sensing platforms, and multimodal signal interpretation.

**Fig. 4:**
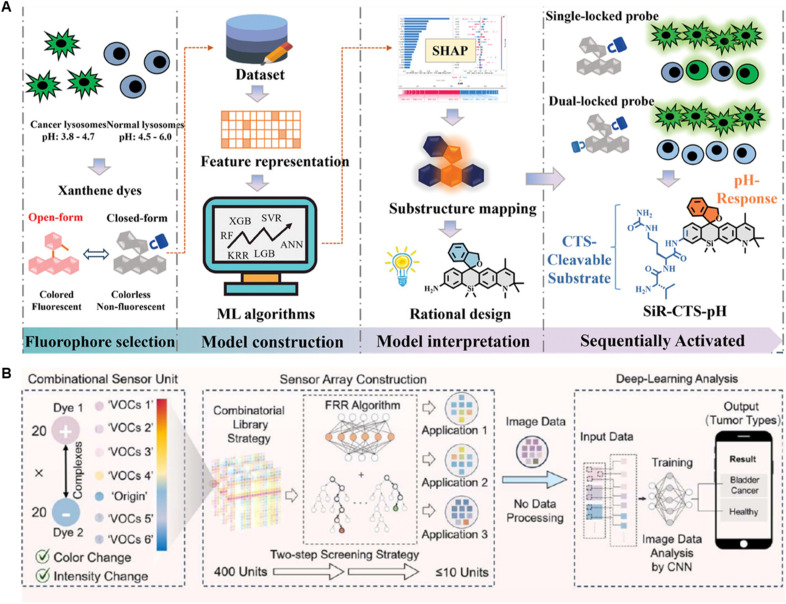
Optimization of analytical procedures and detection platforms using ML. (A) Workflow of ML-assisted novel dye design, including fluorophore selection, model construction, interpretation, and molecular design guidance. Reproduced with permission.^66^ Copyright 2024, John Wiley & Sons. (B) Schematic illustration of a deep learning-assisted two-step screening strategy for identifying the optimal minimal sensor element combinations in a VOC-targeted sensor library. Reproduced with permission.^14^ Copyright 2025, American Chemical Society.

At the molecular probe design level, ML has demonstrated its capability to establish quantitative structure–property relationships, enabling predictive and rational probe design. For instance, ML-assisted molecular design frameworks have been developed for cyanine-based photosensitizers by integrating RDKit structural descriptors with quantum chemical descriptors, allowing accurate prediction of singlet oxygen quantum yield (*Φ*_Δ_) and fluorescence quantum yield (*Φ*_F_) with coefficients of determination exceeding 0.9.^65^ Based on these predictive models, a two-stage virtual screening strategy was applied to a library of 2835 candidate structures, leading to the identification of high-performance molecules subsequently validated experimentally. The lead compound exhibited a *Φ*_Δ_ value of 0.62 and demonstrated favourable performance in cellular assays, validating the reliability of the ML-guided screening workflow for diagnostic applications. Similarly, ML-guided rational design has been applied to xanthene-based fluorescent probes to achieve precise pH responsiveness (Fig. 4A).^66^ By learning from existing dye datasets, predictive models were constructed to guide the synthesis of novel Si-rhodamine derivatives, enabling the development of dual-activated probes responsive to both cathepsin activity and acidic tumour microenvironments. Such probes exhibited superior signal-to-noise ratios and enhanced discrimination capability between tumour and normal tissues in complex biological samples.

Despite these advances, probe-level optimization alone is insufficient for practical cancer diagnostics. Probe performance is highly context-dependent and can be strongly influenced by sensor interfaces, device architectures, and measurement conditions. Moreover, even highly optimized probes do not necessarily translate into optimal diagnostic accuracy at the system level, particularly in heterogeneous clinical samples. These limitations have driven a paradigm shift toward ML-assisted construction and optimization of integrated analytical platforms.

At the platform level, ML has been used to model complex physical and chemical couplings that are difficult to resolve analytically. In microfluidic biosensing systems, hybrid ML models combining artificial neural networks with particle swarm optimization have successfully predicted detection times in sensing devices by accounting for parameters such as rotational speed, angular alignment, and sensor positioning.^67^ Although not originally developed for cancer diagnostics, such approaches demonstrate the general applicability of ML for optimizing biosensor performance. In electrochemical immunosensors for cancer diagnostics,^68^ ML algorithms including *k*NN and SVM have been applied to extract diagnostic information from voltametric features beyond peak current intensity, such as peak potential shifts and peak broadening. Using this strategy, positive samples containing the cancer biomarker p53 were accurately identified in artificial urine and saliva matrices, with detection sensitivities reaching 0.26 ng mL^−1^. Notably, the selection of redox probes was shown to exert a greater influence on diagnostic performance than electrode architecture, underscoring the importance of holistic platform design over isolated hardware optimization. More recently, ML-assisted optimization has been extended to advanced sensor architectures, such as terahertz meta-surface biosensors,^19^ where regression models guide structural parameter design with near-perfect prediction accuracy. Combinatorial sensor array strategies further highlight the advantages of platform-level ML optimization. In volatile organic compound (VOC)-based cancer diagnostics, a feedforward neural network-random forest-recursive feature elimination algorithm-assisted screening of a sensing library comprising 400 elements enabled the identification of minimal arrays containing only 8–10 sensor elements, while maintaining 100% discrimination accuracy between different VOC models (Fig. 4B).^14^ These results demonstrate that ML can substantially reduce system complexity while preserving diagnostic performance, facilitating the development of simplified and portable point-of-care platforms.

As analytical platforms become increasingly sophisticated, they inherently generate high-dimensional and heterogeneous data that exceed the capacity of conventional single-parameter analysis. In this context, ML improves performance not by changing the sensing hardware, but by extracting weak yet complementary information from existing outputs, integrating correlated signals, and learning more informative decision rules. By capturing nonlinear relationships and reducing noise, ML can enhance sensitivity, robustness, and diagnostic accuracy through more effective data interpretation. For example, in lateral flow assay-based cancer diagnostics, synergistic prediction by support vector regression and Gaussian process regression enabled integrated analysis of colorimetric and fluorescence signals, achieving femtomolar level detection of cancer related microRNAs within 5 minutes.^69^ This approach yielded coefficients of determination of approximately 0.99 and supported the simultaneous detection of multiple microRNA targets. Similarly, in multi-resonant biosensing architectures, ML-based integration of multiple optical resonances has been shown to improve detection precision by up to three orders of magnitude compared with single-resonance analysis,^70^ highlighting the power of data-centric strategies for performance enhancement.

Taken together, these developments demonstrate that ML has evolved into a systematic framework spanning molecular design, platform construction, and multimodal data interpretation, enabling the optimization of analytical procedures and detection platforms. This evolution underscores the growing importance of data-driven strategies for overcoming hardware and material limitations, particularly in complex biological samples. Nevertheless, the robustness and generalizability of ML models across different platforms remain to be systematically validated. To advance the field, greater emphasis should be placed on standardized data acquisition and benchmarking strategies to ensure reproducibility and facilitate the translation of ML-assisted diagnostic systems into routine clinical practice.

### 3.2 Interpretation of imaging-based data

The increasing adoption of advanced imaging technologies has profoundly expanded the volume and complexity of data generated in cancer diagnostics. Modern imaging modalities, ranging from histopathology and radiology to emerging nanoscale and spectroscopic techniques, provide rich spatial and phenotypic information but also pose substantial challenges for interpretation due to high dimensionality, variability, and the limited scalability of human analysis. In this context, ML has emerged as a central analytical framework for extracting diagnostically relevant information from imaging data and translating it into clinically actionable insights. Recent progress in ML-based imaging analysis broadly follows a trajectory from algorithmic development and interpretability toward the integration of machine learning with increasingly sophisticated imaging platforms.

Early applications combining ML algorithms with established imaging modalities such as histopathology and radiology have demonstrated strong potential for direct image level disease classification. For example, Cheng *et al.* trained recurrent neural network models on large, well annotated whole slide image datasets and achieved diagnostic performance comparable to that of human experts.^71^ Their ML based cervical cancer screening systems demonstrated sensitivities and specificities of approximately 95% and 93.5%, respectively, across multi centre cohorts, while maintaining clinically feasible inference times of around 1.5 minutes per gigapixel image. Similarly, Tolkach *et al.* applied convolutional NN frameworks to prostate cancer pathology,^72^ enabling tumour detection and Gleason grading with overall accuracies approaching 97–98%, thereby establishing ML as a reliable tool for routine histopathologic assessment.

Beyond image level classification, ML has enabled the extraction of higher order biological information that is not directly accessible through visual inspection. Explainable ML approaches applied to breast cancer histology have shown that morphological features encoded in H&E-stained images can be used to predict molecular characteristics, including DNA methylation patterns, gene expression profiles, copy number variations, and somatic mutations.^73^ These approaches have achieved balanced accuracies of approximately 78%, exceeding 95% in selected patient subgroups. More recently, Hoang *et al.* developed hybrid modelling strategies that explicitly predict molecular intermediates, such as DNA methylation beta values, from histopathology images prior to tumour classification.^74^ In central nervous system tumours, such approaches have achieved overall classification accuracies of approximately 95% on external validation cohorts, highlighting the potential of imaging data to serve as a surrogate for molecular profiling.

Despite these advances, algorithm-centric improvements alone face intrinsic limitations. Model performance remains sensitive to domain shifts introduced by variations in staining protocols, imaging hardware, and acquisition settings. In addition, the biological interpretation of image derived molecular predictions often requires complementary experimental validation. Moreover, the diagnostic information content is ultimately constrained by the imaging modality itself, motivating efforts to enhance both the quality and diversity of image derived signals through the development of novel imaging techniques in conjunction with ML.

**Fig. 5:**
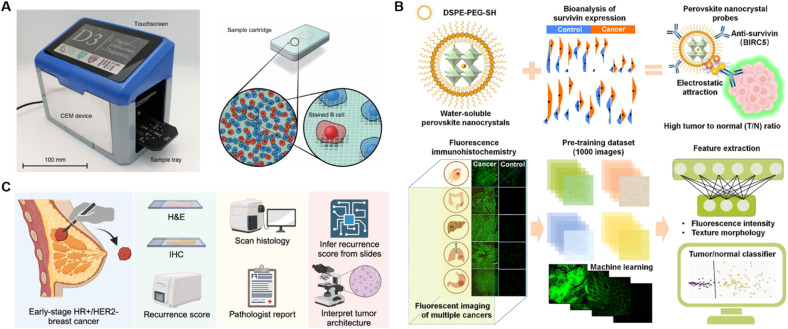
Interpretation of imaging-based data using ML. (A) The image illustrates combined contrast-enhanced microholography integrated with convolutional NN to enable automated analysis of fine-needle aspirates for point-of-care lymphoma diagnosis. Reproduced with permission.^11^ Copyright 2018, Springer Nature. (B) SVM-based framework for fluorescent imaging using perovskite nanocrystal probes for the pathological diagnosis of breast, colon, liver, lung, and stomach cancers. Reproduced with permission.^76^ Copyright 2024, American Chemical Society. (C) Multimodal transformer model-enabled workflow for recurrence risk modelling in early-stage breast cancer. Tumours are surgically resected, histologically profiled, digitally scanned, and analysed for downstream prediction of recurrence risk. Reproduced with permission.^80^ Copyright 2025, Springer Nature.

Recent studies have demonstrated that coupling ML with emerging imaging modalities can substantially expand diagnostic capabilities. For instance, Im *et al.* combined contrast enhanced microholography with deep learning to enable automated analysis of fine needle aspirates for lymphoma diagnosis at the point of care, achieving high classification accuracy in prospective clinical studies involving dozens of patients (Fig. 5A).^11^ At smaller spatial scales, nanoscale imaging approaches have introduced new classes of diagnostically informative features. RF and GBT-based analysis of atomic force microscopy images capturing cell surface nanostructures has achieved diagnostic accuracies of approximately 94% for bladder cancer detection using only a small number of cells per patient,^75^ underscoring the discriminative power of nanoscale morphological information.

ML has also played a critical role in unlocking the diagnostic value of chemically and spectroscopically rich imaging modalities. Chi *et al.* combined high contrast fluorescence imaging using perovskite nanocrystal probes with SVM-based feature extraction from intensity and texture patterns, enabling rapid pathological diagnosis across multiple cancer types (Fig. 5B).^76^ This approach achieved an area under the receiver operating characteristic curve (AUROC) exceeding 0.90 and improved tumour to normal contrast by more than an order of magnitude compared with conventional probes. Similarly, label free photoacoustic microscopy with subcellular resolution, integrated with virtual staining and densely connected convolutional networks-based classification, enabled robust discrimination between malignant and benign tissues, achieving an AUROC of 0.902 and offering a rapid alternative to conventional histological workflows.^77^ In mass spectrometry imaging, artificial NN-guided analysis of spatial lipidomic and elemental distributions enabled accurate differentiation between tumour and adjacent normal tissues, with discovery cohort accuracies reaching 100% and independent validation accuracies exceeding 90%.^78^

To further improve diagnostic performance, multimodal ML approaches that integrate complementary imaging, molecular, and clinical data have been developed to mitigate the limitations of individual modalities and enhance robustness. For example, although low dose computed tomography is widely used for early lung cancer screening, its specificity and sensitivity remain limited. Cai *et al.* presented a multimodal early screening platform that integrates computed tomography imaging with multiplexed protein biomarker data using multivariate logistic regression, achieving an AUROC approaching 0.94 and substantially improving diagnostic accuracy.^79^

Encouragingly, ML-enabled imaging analysis has also demonstrated strong performance in large scale and population-based validation studies. In breast cancer, deep learning models trained on 6172 H&E whole slide images successfully inferred clinically established recurrence scores, achieving an AUROC of approximately 0.89 and outperforming traditional clinicopathologic nomograms (Fig. 5C).^80^ Similarly, deep learning systems applied to non-contrast computed tomography imaging for gastric cancer screening achieved an AUROC of 0.97 in internal validation and 0.93 in multi centre external cohorts comprising tens of thousands of cases.^81^ Notably, these systems identified early-stage cancers missed by routine radiologic interpretation in real world screening scenarios. Together, these studies highlight the potential of ML-enabled imaging diagnostics to be translated into routine clinical practice and to realize their full promise in precision oncology.

### 3.3 Interpretation of molecular profiling data

Nowadays, the development of high-throughput sensing technologies and liquid biopsy platforms has become an important driver reshaping cancer diagnostics. Modern assays increasingly generate complex and high-dimensional molecular and biochemical data, ranging from proteomic and metabolomic fingerprints to genomic, epigenomic, multimodal, and unconventional signals. With the assistance of ML, these heterogeneous and noisy signals can now be transformed into clinically actionable diagnostic outputs more effectively. As summarized in Table 2, different biomarker classes have distinct diagnostic value and methodological challenges, and therefore require tailored ML strategies.

**Table 2:** Categorization of molecular profiling biomarkers and ML strategies in cancer diagnostics

	Typical data	Key value and main challenges	How ML is tailored	Key references
Proteomic biomarkers	Multiplexed protein panels, exosomal markers, immune-related amino acid signatures	Closest to clinical assays and relatively easy to translate, but affected by biological variability and immune-status noise	Pattern decoding and classification are the main goals, so RF, LDA, and weighted classifiers are commonly used	82–84
Metabolomic biomarkers	Serum, urine, and EV-associated metabolic fingerprints	Useful for detecting global metabolic reprogramming, but vulnerable to diet, medication, circadian rhythm, comorbidities, and batch effects	Feature selection and robust classification are the main goals, so sparse regression, SVM, and RF are commonly used	85–92
Genomic and epigenomic biomarkers	Mutations, DNA methylation, microRNA	Strong diagnostic power and interpretability, but often requires careful feature selection due to high dimensionality	Regularized or ensemble models and explainable AI are used to handle small-*n*, large-*p* data and preserve interpretability	93–98
Multimodal biomarkers	Combined protein, gene methylation, and metabolic signals	Improves diagnostic accuracy and robustness, but increases model complexity	Data-fusion or integrated models are used to combine complementary biomarkers and improve robustness	99 and 100
Unconventional biomarkers	Chemically rich spectra, single-particle Raman, microbiome profiles	Expands diagnostic possibilities beyond conventional biomarkers, but standardization and population-level variability remain challenges	Uses RF, SVM, convolutional NN, or large-scale ML to extract latent diagnostic features from non-standard signals	12, 101 and 102

**Fig. 6:**
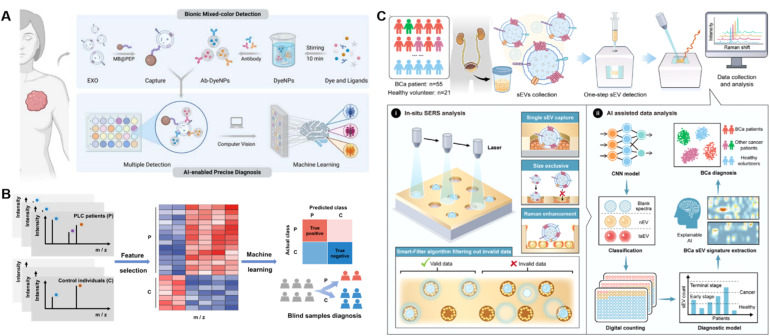
Interpretation of molecular profiling data using ML. (A) Principles of AI-enabled colorimetric analysis of exosome for precise diagnosis of breast cancer. Reproduced with permission.^82^ Copyright 2026, Royal Society of Chemistry. (B) Workflow for the feature selection from mass spectrometry-based fingerprinting data and subsequent machine learning-driven blind sample classification. Reproduced with permission.^92^ Copyright 2023, Royal Society of Chemistry. (C) Schematic illustration of non-invasive bladder cancer diagnosis using a fully integrated single extracellular vesicle isolation platform combined with AI-assisted Raman spectral analysis. Reproduced with permission.^12^ Copyright 2025, John Wiley & Sons.

Progress in this area has primarily focused on protein-related biomarkers, which are conceptually closest to established clinical assays and therefore present a relatively low barrier to clinical translation. Rather than relying on single protein markers, recent studies have demonstrated that ML can decode combinatorial protein patterns that better reflect tumour heterogeneity and host immune responses. A representative example is the ML-enabled bionic mixed-colour sensing technology developed for breast cancer subtyping (Fig. 6A),^82^ in which exosomal surface proteins including PD-L1, EpCAM, and HER2 are simultaneously labelled with color-coded nanoprobes. The resulting mixed-colour fingerprints are captured as intuitive visual signals and decoded using RF, achieving perfect discrimination among cell line models and 96.7% accuracy in clinical samples. In addition, Li *et al.* reported an aptamer-based nanoflow cytometry strategy for rapid profiling of multiple protein markers on small extracellular vesicles.^83^ By applying linear discriminant analysis and RF classifiers, molecular classification of ovarian cancer cell lines and subtypes was achieved with overall accuracies of 82.9% and 55.4%, respectively. Beyond tumour-derived proteins, immunodiagnostic strategies that focus on systemic immune activation have revealed complementary diagnostic information. Tang *et al.* demonstrated that plasma amino acid residue signatures, reflecting cancer-specific immune responses, could be analysed using an ensemble subspace discriminant classifier to detect cancer with an AUROC of 0.95, and to predict therapeutic response in selected patient subsets.^84^ While immune-associated biomarkers improve sensitivity to early disease states, they also introduce additional variability related to infection, inflammation, and individual immune status, highlighting the importance of uncertainty estimation and longitudinal sampling in ML-driven diagnostic models.

As the field has matured, attention has increasingly shifted from predefined biomarker panels toward holistic molecular fingerprints, particularly in metabolomics. These approaches view cancer as a systemic metabolic reprogramming process rather than a collection of isolated molecular events. ML is particularly well suited to this paradigm, as it can identify stable disease-associated patterns from large numbers of weakly specific and noisy features. For example, early-stage lung adenocarcinoma has been diagnosed with sensitivities of approximately 70–90% and specificities of 90–93% using sparse regression analysis of metabolic patterns acquired by laser desorption ionization (LDI) mass spectrometry.^85^ Pan-cancer strategies such as multiplexed nanomaterial-assisted LDI mass spectrometry further demonstrate how machine learning can integrate complementary metabolic fingerprints to enable both cancer detection and tissue-of-origin classification across large cohorts.^86^ Similar frameworks have been successfully applied to cancers of the oral cavity,^87^ breast,^88^ stomach,^89,90^ bladder,^91^ and liver (Fig. 6B),^92^ using serum, urine, or extracellular vesicle-associated metabolic or glycomic profiles, and often achieving AUROC exceeding 0.9 in validation cohorts.

Despite their promise, metabolomic and glycomic diagnostics also expose fundamental limitations. Metabolic profiles are highly sensitive to confounding factors such as diet, medication, circadian rhythms, and comorbidities, which can obscure disease-specific signals and reduce model transferability across populations. Many studies report strong performance in discovery cohorts but show attenuation in external validation, highlighting risks of cohort bias and overfitting. Moreover, the biological interpretability of metabolic fingerprints remains limited, as ML models often rely on complex combinations of features that do not map directly onto known pathways. Addressing these challenges will require stricter control of pre-analytical variables, incorporation of biologically constrained feature selection, and integration with orthogonal data types to improve robustness.

In parallel, genomic and epigenomic biomarkers have emerged as some of the most powerful data sources for cancer diagnosis. Nguyen *et al.* developed a whole-genome mutation-based RF classifier that leverages both driver and passenger mutation patterns to resolve tissue of origin in cancers of unknown primary with high precision and recall, while maintaining feature-level interpretability.^93^ DNA methylation-based classification has demonstrated even greater clinical impact. The landmark work by Capper *et al.* established genome-wide methylation profiling as a diagnostic standard for central nervous system tumours, improving diagnostic precision and leading to changes in diagnosis in up to 12% of prospective cases.^94^ Subsequent studies have addressed limitations related to interpretability and platform dependence. Explainable AI frameworks developed by Benfatto *et al.* revealed that methylation classifiers rely on distributed yet biologically meaningful genomic regions, enhancing robustness and clinical trust.^95^ More recently, the crossNN machine learning framework enabled accurate tumour classification from sparse methylomes generated across diverse sequencing platforms, achieving precisions of 99.1% for brain tumours and 97.8% for pan-cancer classification.^96^ In addition, microRNA signatures combined with ML have shown promising diagnostic performance, particularly in estrogen receptor-positive breast cancer and colorectal cancer.^97,98^

Despite these advances, no single class of biomarkers is sufficient to meet the requirements of precise cancer diagnosis. To address this limitation, Fedyuk *et al.* developed a single-molecule multiparametric assay, EPINUC, which integrates epigenetic profiling of plasma-isolated nucleosomes with DNA methylation and cancer-specific protein biomarkers.^99^ Using a logistic regression model, this approach achieved an AUROC of 0.96 with sensitivities of up to 92% at high precision. Similarly, Yang *et al.* combined urine metabolic fingerprints with urine protein levels and sparse learning to construct a diagnostic model capable of accurately characterizing kidney disease subtypes.^100^

Beyond conventional molecular assays, ML has also unlocked diagnostic value from unconventional data types, including chemically rich spectra, single-particle measurements, and microbiome profiles. Spectral fingerprinting using quantum-defect-modified carbon nanotubes produces near-infrared fluorescence patterns that, when analysed using ML algorithms such as RF and SVM, enable ovarian cancer detection with sensitivity and specificity exceeding established serum biomarkers.^101^ Single extracellular vesicle Raman mapping further demonstrates that convolutional NN can extract diagnostically informative nanoscale features from individual particles, enabling highly accurate early detection of bladder cancer (Fig. 6C).^12^ Microbiome-based diagnostics represent another orthogonal dimension of data-driven cancer detection. Large-scale ML analysis of faecal metagenomic datasets has enabled multi-disease classification across diverse phenotypes with high accuracy.^102^ However, strong dependence on geographic, dietary, and lifestyle factors introduces substantial variability, necessitating population-specific calibration before such models can be reliably deployed in clinical practice.

Despite impressive progress, significant barriers remain for routine clinical deployment of ML-driven, data-centric diagnostics. Model performance is often sensitive to cohort composition and pre-analytical variability, and many studies lack large-scale prospective validation. In addition, increasing model complexity raises concerns regarding interpretability and clinical trust. We anticipate that addressing these challenges will require standardized data acquisition protocols, transparent modelling strategies, and rigorous multi-centre validation in populations. Looking forward, the most impactful advances are likely to emerge from the convergence of robust sensing technologies, biologically informed machine learning models, and clinically grounded validation frameworks.

## 4 Challenges and perspectives

### 4.1 Grand challenges

ML has demonstrated substantial potential in improving the precision of cancer diagnostics. The development of ML-based diagnostic systems is inherently interdisciplinary and integrates data science, analytical chemistry, materials science, bioengineering, and clinical medicine. Despite these advances, several key challenges remain across the entire pipeline. These challenges include limitations in data quality and quantity, lack of standardization, and concerns regarding model bias and generalizability. In the following sections, we will discuss these challenges and advances which have been achieved to address them.

#### 4.1.1 Data quality and quantity

ML models require large, high-quality, and well-annotated datasets to learn robust and generalizable patterns. However, acquiring such datasets remains challenging in cancer diagnostics. Many high-performance studies rely on discovery cohorts with limited sample sizes. This issue is particularly pronounced in omics-based studies, where the number of extracted features often far exceeds the number of samples. Under these conditions, models are prone to overfitting and may fail to capture the variability present in real-world clinical populations.^25^ In addition, as mentioned earlier, features from metabolomics, glycomics, or microbiome data are sensitive to diet, collection time, storage conditions, and extraction protocols. When these variables are not systematically recorded, it becomes difficult to account for their effects during modelling. As a result, models that achieve excellent internal AUROCs often show decreased performance in external validation cohorts.

Another concern relates to the reliability of reference labels used for model training. In clinical practice, diagnoses based on H&E-stained images may vary between observers, introducing label noise. The development of DNA methylation-based classifiers provides an example of how ML-based molecular profiling can improve diagnostic consistency.^94^ Notably, these classifiers allow a “no match” result when a tumour does not confidently correspond to predefined categories, thereby reducing forced misclassification. In prospective evaluation, the method led to a change of diagnosis in up to 12% of cases, highlighting its impact on diagnostic precision.

To mitigate these limitations, larger prospective and multi-centre cohort studies are needed. Training and validation cohorts should include balanced case–control ratios and representative stage distributions. Comprehensive recording of pre-analytical metadata, such as sampling time, diet, medication use, storage conditions, and device calibration, is essential. These variables can be incorporated as covariates or used for stratified analyses to reduce confounding effects. Although this may increase clinical workload, it is valuable for building reliable ML-based diagnostic systems and guiding future development.

#### 4.1.2 Lack of standardization

Heterogeneity in assays, platforms, and preprocessing pipelines creates data shifts that reduce ML model portability. Unlike imaging data, which usually follow a more common structure, many molecular and sensor-based assays lack universal standards. For example, nanomaterials, spectrometers, sequencing platforms, and microfluidic devices used across studies may differ in size, sensitivity, and dynamic range, leading to platform specific signal distributions. Although cross platform approaches such as crossNN and related methylation-based calibration strategies have attempted to address these discrepancies,^94,96^ their applicability remains limited. In addition, preprocessing pipelines also vary widely. Steps such as peak detection, normalization, deconvolution, and feature selection strongly affect downstream modelling. Inconsistent preprocessing across laboratories is a major source of irreproducibility. Consequently, models trained in one centre or on one platform may fail during deployment, slowing clinical translation and increasing regulatory challenges.

Improving standardization requires coordinated efforts at multiple levels. First, standard operating procedures should be established and publicly documented for sample collection, handling, instrument calibration, and data preprocessing. The use of internal standards and spike in controls should be encouraged whenever feasible. Second, open source and containerized preprocessing pipelines, for example, implemented in reproducible workflow systems such as Nextflow,^103^ should be provided with explicit parameter settings to ensure reproducibility. Transparent reporting of cross-validation strategies and hyperparameter tuning is also essential. Finally, further development of cross platform calibration is necessary to enhance model transferability.

#### 4.1.3 Model bias and generalizability

ML models can encode and amplify biases present in training data, leading to unequal performance across demographic groups. Training cohorts often underrepresent minority populations, specific age groups, or patients with certain comorbidities. As a result, a model that performs well in a discovery cohort may underperform in underrepresented populations. For example, microbiome signatures vary according to diet and geography, which may cause models trained in one region to misclassify samples from another.^102^ Therefore, careful model selection and validation are essential in studies where specific biases are known to exist.

Another important issue concerns explainability and clinical trust. Black box models are more difficult to translate into clinical practice because clinicians need to understand which features drive the prediction and whether the output is biologically plausible. Although substantial progress has been made in developing explainable approaches to help identify the contributions of individual variables or image regions, including SHapley Additive exPlanations (SHAP),^104^ attention mechanisms,^105^ and other feature attribution techniques,^106^ many of these methods still require validation in larger and more diverse cohorts.

Data level strategies to improve generalizability have been discussed above. At the model level, performance should be routinely evaluated across demographic subgroups, and disparities should be explicitly reported. When necessary, methods such as reweighting or domain adaptation can be applied to mitigate distributional differences. In addition, models should provide well-calibrated prediction probabilities along with uncertainty estimates, enabling clinicians to interpret outputs more cautiously. Cases with low confidence can be flagged for further review or additional testing to reduce the risk of inappropriate clinical decisions. Finally, when feasible, hybrid or interpretable models that support feature attribution are preferable, as they enhance transparency and facilitate clinical trust and accountability.

### 4.2 Future directions

Despite the grand challenges in the above section, ML reveal merits and advantages in the clinical diagnostics. Future development of this area will not only address existing challenges but also demonstrate their potential in compiling multidisciplinary technologies.

#### 4.2.1 Multimodal data integration

No single data modality is sufficient to fully capture the biological complexity of cancer. In clinical practice, diagnosis often relies on a combination of imaging, histopathology, molecular profiling, and laboratory tests to improve accuracy. This creates a need for ML frameworks capable of integrating multimodal data sources. In general, two main strategies are currently used for multimodal integration. One approach involves early fusion,^107^ in which features extracted from different modalities are combined into a unified representation before model training. The other approach relies on late fusion, where modality specific models are trained separately and their outputs are combined at the decision level.^108,109^ Early fusion may capture cross modality interactions more directly, but it is sensitive to missing data and differences in feature scale. Late fusion is often more flexible and modular, yet it may fail to model complex biological interactions across modalities.

Despite increasing interest in multimodal ML for cancer diagnostics, several challenges limit clinical implementation. First, different modalities operate at distinct spatial and temporal scales and have heterogeneous data structures. Imaging data are typically high dimensional and spatially structured, whereas omics data are often sparse. Harmonizing these heterogeneous representations into a coherent analytical framework remains difficult. Second, the dynamic ranges of different data types vary substantially. For example, genomic alterations may be binary or categorical, while metabolomic measurements span continuous concentration ranges. Models must account for these differences without allowing one modality to dominate the learning process. Third, missing data are common in real world clinical settings. Not all patients undergo the same imaging protocols or molecular tests. Many current multimodal models assume complete data, which limits their applicability. Robust strategies for handling partially observed modalities are therefore required.

Future efforts should focus on developing standardized frameworks for multimodal data harmonization, including cross platform normalization and structured metadata recording. Model architectures that are robust to missing modalities and capable of quantifying uncertainty should be prioritized.

#### 4.2.2 Human-in-the-loop system

Clinical diagnostics require transparency and accountability. Fully automated systems without interpretable reasoning may limit clinician acceptance. Human-in-the-loop systems aim to combine algorithmic prediction with expert oversight.^110^ In such frameworks, ML models assist clinicians by prioritizing suspicious cases, highlighting relevant regions, or generating structured risk estimates, while the final decision remains under human control. However, the optimal design of interaction mechanisms remains unclear and challenging to define. Looking forward, systems that provide useful guidance without increasing cognitive load or generating excessive alerts are likely to be particularly valuable.

Many explainable AI methods have been developed to clarify model behaviour through feature attribution or simplified surrogate models, which show potential for incorporation into human-in-the-loop systems. Although these approaches can increase transparency, many explanations remain approximations and do not ensure causal interpretability. Future efforts should emphasize robust and reproducible explanation methods and evaluate whether interpretability meaningfully improves diagnostic performance and trust in prospective clinical studies.

#### 4.2.3 Active and transfer learning

The development of ML models for cancer diagnostics is often constrained by limited labelled data. Annotation typically requires expert interpretation, molecular validation, or longitudinal follow up, which increases cost and time. Active learning and transfer learning provide strategies to improve data efficiency under these constraints.

Active learning reduces annotation burden by prioritizing samples that are expected to provide the greatest performance gain, such as cases with high predictive uncertainty.^111^ Although this approach has shown promise in imaging and rare tumour classification, its integration into clinical workflows remains limited. Reliable uncertainty estimation and streamlined expert feedback mechanisms are required to make active learning practically feasible.

Transfer learning leverages pretrained models to improve performance on related diagnostic tasks.^112^ While fine tuning pretrained models can enhance performance and reduce training time, domain shift between source and target datasets often limits generalizability. Future work should systematically evaluate transferability across institutions and cancer types and develop adaptation strategies that explicitly account for distributional differences.

We anticipate that additional practical and easy-to-implement strategies, including active and transfer learning, will be developed to further reduce annotation demands and related burdens in cancer diagnostics. Future efforts should prioritize reproducibility, cross-institutional validation, and integration with existing clinical data systems to enhance real-world applicability.

#### 4.2.4 LLM-assisted diagnostics

Beyond conventional predictive models, large language models (LLMs) are emerging as a new interface between complex diagnostic data and clinical reasoning. Unlike traditional task-specific ML classifiers, which typically return a probability or category, LLMs can integrate heterogeneous information from pathology reports, imaging descriptions, molecular test results, and clinical notes into structured diagnostic interpretations.^113^ This capability may be particularly valuable in future cancer diagnostics, where decision-making often depends on combining fragmented evidence across multiple clinical domains. In addition, we believe translating model outputs into clinically meaningful explanations could significantly improve communication among radiologists, pathologists, oncologists, and patients.

However, LLM-based diagnostic interpretation also introduces distinct challenges. First, LLMs may generate plausible but incorrect explanations, particularly when the underlying evidence is incomplete or ambiguous. Second, sensitive clinical information requires strict privacy protection and careful governance. Balancing the need for sufficient information to support reliable interpretation with the need to protect donor privacy will be essential for safe and effective use. Future efforts should therefore focus on robust validation frameworks and privacy-preserving methods to support clinical deployment.

#### 4.2.5 POCT devices

Point-of-care testing (POCT) refers to diagnostic testing performed near the patient, rather than in centralized laboratories. The World Health Organization has outlined key requirements for POCT, including affordability, sensitivity, specificity, user-friendliness, rapid turnaround time, robustness, minimal equipment dependence, and accessibility to end users.^114^ POCT devices are particularly valuable in resource-limited settings and for routine cancer screening or monitoring of slowly progressing diseases. However, data generated by POCT devices often exhibit higher variability and lower resolution compared with laboratory-based systems. ML methods applied to these data through smartphone-assisted analysis may help improve diagnostic accessibility and consistency. To be effective in this context, ML models must be robust to noise, limited computational capacity, and constrained energy resources.

Future research should focus on lightweight and resource-efficient model architectures suitable for edge deployment. Standardized validation under real-world operating conditions is necessary to ensure reliability and safety. Close coordination among engineering development, clinical evaluation, and regulatory oversight will be essential for responsible implementation.

#### 4.2.6 Regulatory pathways

The clinical translation of ML-based cancer diagnostics is significant, but it will depend on the establishment of clear and adaptive regulatory pathways. Unlike conventional diagnostic tests, ML-based systems may rely on continuously updated datasets or adaptive algorithms, so their performance at deployment may differ from that of the version initially validated. This means that premarket evaluation alone is unlikely to be sufficient, and future regulation will need to address the full life cycle of these diagnostic tools. This will include rigorous external validation across multiple sites and strengthened post-market surveillance to detect performance drift. In addition, ethical considerations in the use of ML may further shape regulatory pathways by requiring greater transparency, accountability, and bias mitigation. We foresee that addressing these issues will require clear standards for data quality and representativeness, as well as routine bias auditing, ongoing human oversight, and the development of more clinically interpretable models.

## 5 Conclusions

In summary, ML has become an important analytical framework in cancer diagnostics. We outlined major supervised ML algorithms and discussed their practical applications across different scenarios, highlighting their respective strengths and limitations. We further summarized the use of ML in analytical workflow optimization, as well as in the interpretation of imaging-based and molecular profiling data. Across these domains, ML has demonstrated utility in improving signal extraction, pattern recognition, and multi-scale data integration.

Despite these advances, several challenges still remain. Data-related issues, including limited labelled datasets and inter-institutional heterogeneity, continue to constrain model development and external validation. Methodological limitations, such as varied preprocessing pipelines, and lack of standardization, affect robustness and clinical trust. At the model level, bias, limited generalizability, and barriers to real-world deployment must be systematically addressed before widespread clinical adoption. Future development could focus on six key directions. First, multimodal learning approaches that integrate imaging, molecular, and clinical data may improve diagnostic accuracy and biological insight. Second, human-in-the-loop systems can enhance collaborative decision-making by combining algorithmic predictions with expert oversight. Third, data-efficient strategies, including active learning and transfer learning, are needed to reduce annotation burden and improve cross-institutional adaptability. Fourth, LLM-assisted diagnostics may help translate complex fragmented information into clinically meaningful interpretations and improve communication among clinicians and patients. Fifth, integrating ML into POCT devices may expand access to cancer diagnostics, particularly in resource-limited settings, provided that models are optimized for robustness and edge deployment. Finally, clearer regulatory pathways will be essential to support validation, post-market surveillance, and lifecycle governance of adaptive ML-based tools. We anticipate that ML-empowered cancer diagnostics will increasingly emerge in clinical settings at an unprecedented pace and become more reliable, interpretable, and clinically actionable in real-world practice.

## Author contributions

Both authors contributed to the conception, writing, and revision of the manuscript and approved the final version.

## Conflicts of interest

There are no conflicts to declare.

## Data Availability

No primary research results, software or code have been included, and no new data were generated or analysed as part of this review.
